# Beyond Resistance: Tolerance and Resilience of Bacteria to Photodynamic and Oxidative Stress

**DOI:** 10.3390/ijms26188908

**Published:** 2025-09-12

**Authors:** Aleksandra Rapacka-Zdonczyk

**Affiliations:** Laboratory of Photobiology and Molecular Diagnostics, Intercollegiate Faculty of Biotechnology, University of Gdansk and Medical University of Gdansk, Abrahama 58, 80-307 Gdansk, Poland; aleksandra.rapacka-zdonczyk@ug.edu.pl

**Keywords:** adaptation, tolerance, resilience, antibacterial blue light, antimicrobial photodynamic inactivation, oxidative stress, cross-stress protection, reactive oxygen species

## Abstract

The increasing reliance on light-based antimicrobial technologies, such as antimicrobial blue light (aBL) and antimicrobial photodynamic inactivation (aPDI), underscores the urgent need to comprehend bacterial survival strategies beyond conventional resistance. Two key phenotypes—tolerance and resilience—have emerged as critical but often conflated mechanisms by which bacteria withstand oxidative and photodynamic stress. While tolerance refers to delayed bacterial killing without changes in MIC, resilience encompasses the active restoration of cellular function after transient stress exposure. Both phenomena may impair treatment outcomes and contribute to long-term persistence, even in the absence of genetic resistance. This review dissects the molecular mechanisms underlying tolerance and resilience, with a focus on their relevance to bacterial responses to reactive oxygen species generated by light-based or chemical stressors. The regulatory and effector overlap between these phenotypes is examined, including antioxidant defense systems, DNA repair pathways, and metabolic rewiring. Furthermore, the role of phenotypic heterogeneity and cross-stress protection in blurring the boundary between survival and recovery is discussed, highlighting challenges in experimental interpretation. Finally, the implications of these adaptive strategies are evaluated in the context of antimicrobial efficacy and safety, with an emphasis on kinetic assays and multidimensional profiling as tools to capture complex treatment outcomes. Clarifying the distinction between tolerance and resilience may help guide the development of robust and evolutionarily stable antimicrobial phototherapies.

## 1. Introduction

The rising failure of antibiotic therapies has intensified interest in non-conventional antimicrobials. Among them, light-based strategies such as antimicrobial photodynamic inactivation (aPDI) and antibacterial blue light (aBL) are particularly attractive because they generate reactive oxygen species (ROS) that inflict multi-target oxidative damage [1,2,3,4,5,6,7,8,9,10,11,12,13]. By acting through photochemical rather than specific molecular targets, these modalities may circumvent classical genetic resistance [14].

Yet bacterial survival under oxidative and photodynamic stress is more nuanced than simple killing. Sublethal or intermittent exposure to aBL or aPDI can trigger phenotypic adaptations that, although not involving resistance in the classical sense, still promote treatment failure and long-term survival [15,16,17,18]. Two such non-genetic strategies are tolerance and resilience.

Tolerance refers to the ability of bacteria to survive lethal conditions without an increase in minimal inhibitory concentration (MIC)—typically through slowed metabolism, biofilm protection, or persister formation [19,20,21,22]. Resilience, in contrast, reflects the capacity to regain physiological function and resume growth after stress removal, emphasizing recovery rather than endurance [23,24]. Although often conflated, these phenotypes embody distinct survival logics: tolerance delays killing, while resilience accelerates regrowth.

In addition, persistence represents yet another distinct phenomenon: a reversible state of dormancy adopted by a small subpopulation of cells that remain non-growing while the stressor is present. Unlike tolerant populations, which survive through slowed metabolic activity, or resilient populations, which recover rapidly after stress removal, persisters simply “wait out” the treatment in a quiescent state [20,21]. Once the antimicrobial challenge is lifted, they can resume growth without having acquired resistance mutations. This distinction is critical, as persistence explains transient treatment failures, while resilience explains how populations rebound after damage [20,23,24].

**Figure 1:**
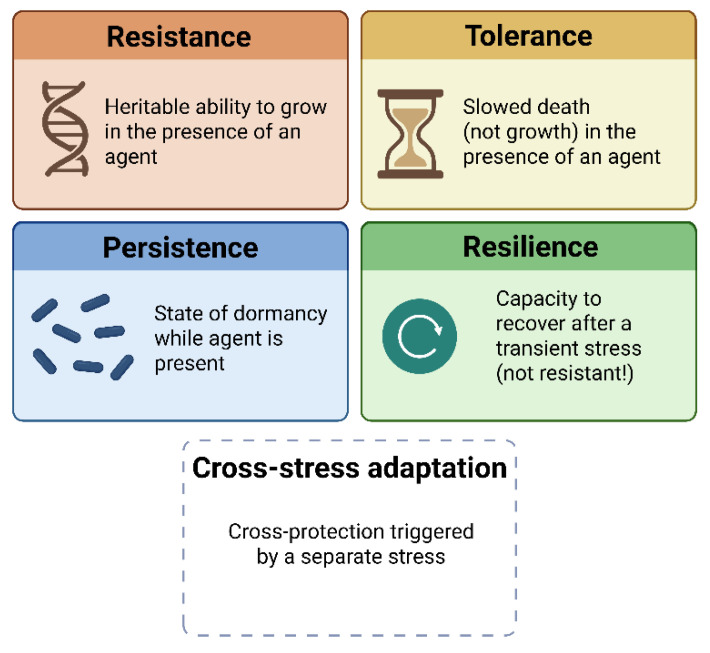
Conceptual distinctions between bacterial survival phenotypes under stress. Resistance refers to the heritable ability to grow in the presence of an antimicrobial agent, whereas tolerance describes a slowed killing rate without changes in MIC. Persistence is a subpopulation-level phenomenon characterized by dormancy in the presence of an agent. Resilience denotes the capacity of bacteria to recover and resume growth after transient stress exposure. Cross-stress adaptation represents cross-protection triggered by exposure to a different stressor.

Together with resistance, persistence, and cross-stress adaptation, these traits form a spectrum of bacterial survival strategies (Figure 1). However, the specific distinction between tolerance and resilience has been largely overlooked—posing both conceptual and experimental challenges that this review aims to address.

Understanding the molecular underpinnings and phenotypic manifestations of both processes is essential for designing effective antimicrobial strategies [25]. In the context of light-based therapies, it remains unresolved whether photodynamic treatments predominantly elicit tolerance, resilience, or both. The overlap between these phenotypes complicates experimental discrimination [23,24]. Moreover, cross-stress protection—where exposure to one stressor enhances survival under another—further obscures interpretation, particularly in multifactorial environments such as infected tissues or food-processing surfaces [26,27,28,29,30,31].

Given the expanding interest in photodynamic approaches and the growing recognition of bacterial plasticity under oxidative pressure, this review aims to:

i clearly define and differentiate tolerance and resilience in the context of ROS-generating treatments;
ii. outline their molecular and regulatory mechanisms;
iii. highlight areas of mechanistic overlap and cross-protection;
iv. discuss the implications of these traits for the efficacy and design of next-generation photonic antimicrobial interventions.

## 2. Photodynamic and Oxidative Stress in Bacteria

**Figure 2:**
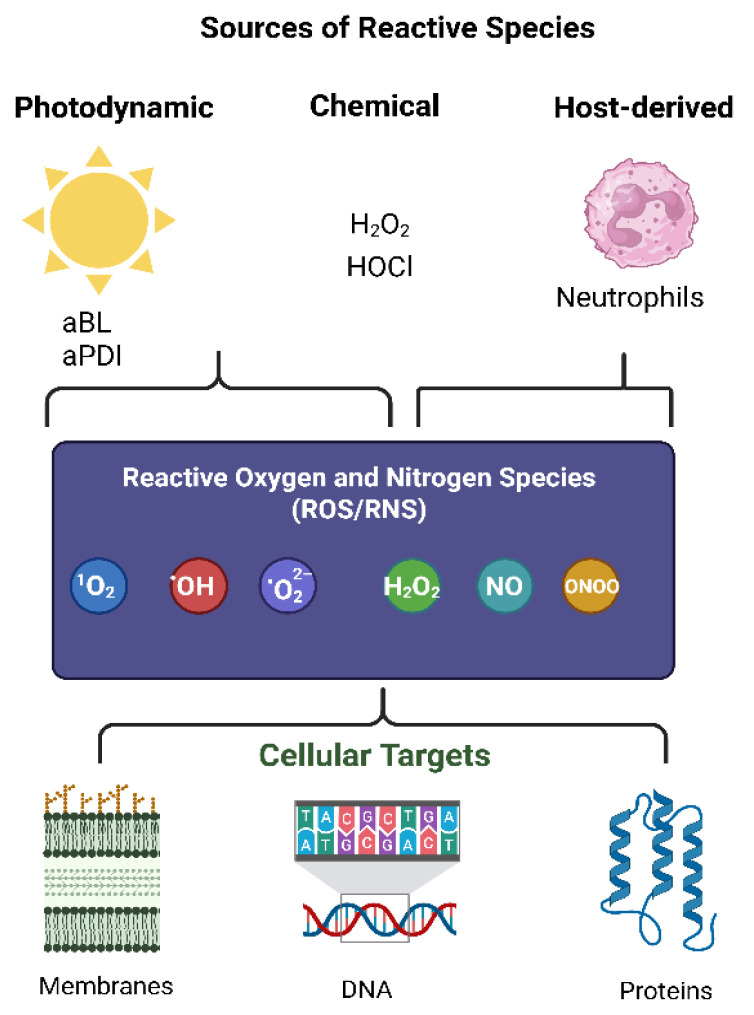
Sources of reactive species and their cellular targets. Photodynamic inactivation (aBL, aPDI), chemical oxidants (e.g., H_2_O_2_, HOCl), and host-derived immune responses (e.g., neutrophils) generate reactive oxygen and nitrogen species (ROS/RNS) such as singlet oxygen (^1^O_2_), hydroxyl radical (•OH), superoxide (•O_2_^−^), hydrogen peroxide (H_2_O_2_), nitric oxide (NO), and peroxynitrite (ONOO^−^). These species attack cellular membranes, DNA, and proteins, leading to oxidative damage and stress responses [12,13,34,35].

Bacteria are constantly exposed to ROS, both from endogenous metabolic processes and external environmental sources. In natural and clinical settings, ROS may arise from the host immune system (e.g., neutrophilic oxidative bursts), environmental oxidants, disinfectants, or antibiotic therapies [25,32]. Additionally, light-based antimicrobial strategies such as aPDI and aBL act by inducing the formation of singlet oxygen (^1^O_2_) and other ROS through the excitation of endogenous or exogenous photosensitizers. In addition to ROS, several stressors and immune defenses generate reactive nitrogen species (RNS), such as nitric oxide (NO) and peroxynitrite (ONOO^−^), which further exacerbate damage to bacterial macromolecules and contribute to nitrosative stress [33]. For instance, activated macrophages and neutrophils produce NO via inducible nitric oxide synthase (iNOS), while acidified nitrite in phagosomes and nitrosylated compounds released at infection sites also serve as potent RNS sources [33,34,35] (Figure 2).

The photodynamic stress elicited by aBL or aPDI typically involves the excitation of porphyrin-like chromophores, leading to electron transfer or energy transfer reactions that generate a spectrum of ROS, including superoxide anion (O_2_^−^), hydrogen peroxide (H_2_O_2_), hydroxyl radicals (•OH), and singlet oxygen (^1^O_2_). These species cause non-specific, multi-target damage to membranes, proteins, nucleic acids, and essential enzymes [12,13]. Oxidative stress may also be triggered by treatment with H_2_O_2_, hypochlorous acid (HOCl), peroxynitrite (ONOO^−^), or reactive halogen species (RHS) produced by host immune defenses [34,35]. Notably, many antibiotics (e.g., fluoroquinolones, aminoglycosides) indirectly cause oxidative stress by disturbing bacterial redox homeostasis and respiratory metabolism [36].

Despite shared effectors (ROS), photodynamic and oxidative stresses differ in their dynamics, localization, and intensity. For example:

Photodynamically generated ROS, especially ^1^O_2_, have very short half-lives and act locally near chromophores, often targeting membranes and periplasmic proteins [37,38].

In contrast, chemically induced ROS, such as H_2_O_2_, can penetrate into the cytoplasm and cause DNA damage or activate stress-responsive regulons, such as OxyR [39,40].

Understanding the sources, chemical nature, and subcellular localization of oxidative stress is critical when investigating bacterial survival strategies. These factors influence the type of cellular damage incurred and the subsequent adaptive responses bacteria deploy [41]. Such responses may tip the balance between tolerance, resilience, and irreversible cell death [19,20,21,22,23].

## 3. Mechanisms of Tolerance

Bacterial tolerance refers to the transient ability to survive lethal stress without a corresponding increase in MIC. Unlike resistance, which is heritable and affects growth in the presence of antimicrobials, tolerance is a phenotypic adaptation that manifests as a delay in bacterial killing. This kinetic delay is best captured by time-dependent metrics such as the minimum duration for killing (MDK), which quantifies the time required to reduce a bacterial population by a defined fraction (e.g., MDK_99_). Tolerant populations often display elevated MDK values while maintaining unchanged MICs, reflecting survival without resistance [20,42,43,44,45].

A hallmark of tolerance is the reduction in metabolic activity, which diminishes the efficacy of treatments that rely on active cellular processes for killing. Within tolerant populations, a subpopulation of slow-growing or dormant cells, known as persisters, often underlies the phenomenon of biphasic killing curves, where a fraction of cells survives treatment much longer than expected under bactericidal conditions. Persisters are reversible phenotypic variants that can resume growth after stress removal and are commonly associated with antibiotic therapy failure and infection recurrence, particularly in biofilm-associated environments [46,47,48,49,50,51].

Mechanisms contributing to tolerance include:

Energy metabolism modulation: Under stress, bacteria may downregulate ATP-consuming biosynthetic pathways while redirecting resources toward protective functions such as efflux activity. Efflux systems transiently lower intracellular concentrations of antibiotics or photosensitizers, thereby reducing treatment efficacy. Because efflux is energetically demanding, these shifts represent a metabolic strategy that promotes transient tolerance [52,53,54,55]. Consistently, pharmacological inhibition of efflux pumps sensitizes *Staphylococcus aureus* biofilms to photodynamic inactivation, underscoring their role in short-term protection [56].

Induction of antioxidant enzymes: Bacteria deploy enzymatic scavengers such as catalases (KatG), superoxide dismutases (SodA), and peroxidases (AhpC) to detoxify ROS and mitigate lethal damage. These protective systems delay killing and protect biomolecules during stress exposure [40,41,57,58]. In contrast, light-based therapies exploit photogenerated ROS—including singlet oxygen and other highly reactive species—against which enzymatic defense is often insufficient, especially in high-flux conditions [37].

Biofilm formation: The extracellular polymeric matrix of biofilms limits the diffusion of antimicrobials and light, creating nutrient-limited zones with low metabolic rates. These conditions favor the survival of tolerant and persister cells [49,59,60,61,62].

Persister cells and stress-regulated dormancy: A subpopulation of tolerant cells, termed persisters, survives lethal treatment by entering a dormant or metabolically inactive state. This phenotype is regulated by global stress-response networks, including the general stress sigma factor RpoS, the alarmone (p)ppGpp, and multiple toxin–antitoxin (TA) modules, which are activated under oxidative or antibiotic pressure. These systems reduce metabolic activity, downregulate ROS-generating pathways, and promote cellular quiescence, thereby limiting the efficacy of ROS-based killing mechanisms. Persisters are not genetically resistant but can endure treatment by effectively “shutting down” susceptible processes until stress subsides. Their formation is enhanced in structured communities such as biofilms but can also arise in planktonic cultures under nutrient limitation or oxidative stress [20,21,42,45,46,47,48,49,50,51,52,57,58,59,60,61,62,63,64,65,66,67,68,69,70].

DNA repair systems (SOS response): Activation of RecA–LexA-dependent repair pathways stabilizes the genome under genotoxic stress. By halting replication and initiating DNA repair (e.g., via UmuD, RecBCD), cells gain time to survive otherwise lethal damage. This system is particularly relevant under ROS-generating treatments such as aBL or aPDI [16,71,72,73,74,75,76,77].

Chaperones and proteases: Under oxidative or proteotoxic stress, bacteria upregulate chaperones like GroEL, DnaK, and disaggregases such as ClpB. These proteins prevent misfolding and aggregation, maintaining proteostasis during exposure. ATP-dependent proteases like ClpXP degrade irreversibly damaged proteins, limiting cellular injury. In this context, chaperone and protease systems primarily function to stabilize protein structures and buffer damage, allowing cells to persist through stress without active recovery [78,79,80,81,82,83].

Envelope stabilization: Tolerant cells may reinforce or modify their outer membrane to limit stressor penetration. Envelope stress response (ESR) systems detect and repair ROS-induced membrane damage, preserving bacterial homeostasis [84,85]. Outer membrane (OM) proteins such as LptD, BamA, and the Mla pathway maintain OM integrity and asymmetry under oxidative challenge, indirectly reducing aBL-induced damage [86,87,88,89].

Metabolic rerouting: Under oxidative or photodynamic stress, bacteria often downregulate ATP-consuming biosynthetic pathways while redirecting carbon flux toward the pentose phosphate pathway (PPP), enhancing NADPH generation to fuel antioxidant defenses. In parallel, glycolysis and the TCA cycle may be suppressed to lower NADH levels and thereby reduce ROS formation from respiratory activity. Such redox reprogramming supports survival during exposure by limiting endogenous ROS and providing reducing power for detoxification and repair. Recent work also shows that pathogens can reconfigure glycolytic flux to maintain proton motive force and redox homeostasis under immune-derived oxidative stress [53,54,90,91].

**Figure 3:**
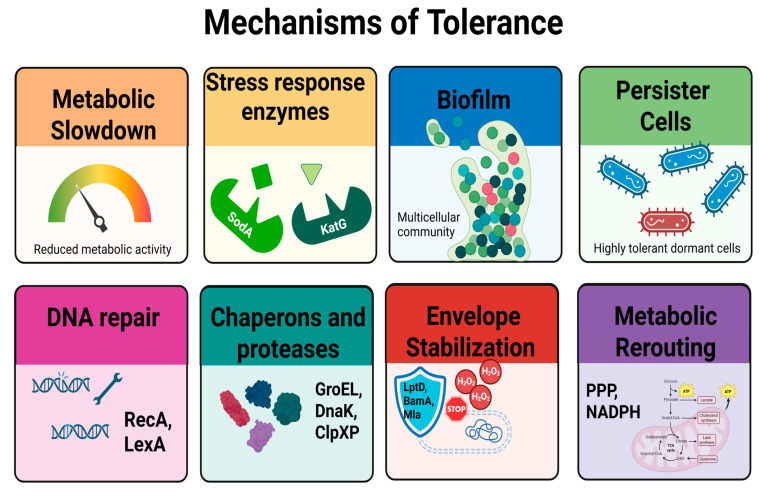
Mechanisms underlying bacterial tolerance to antimicrobial stress. Tolerance is a transient, non-heritable phenotype characterized by delayed killing in the presence of lethal agents. Key mechanisms include metabolic slowdown, which limits the activity of treatments targeting active processes; induction of antioxidant enzymes such as catalase (KatG) and superoxide dismutase (SodA); formation of protective biofilms; and the presence of persister cells, which are metabolically quiescent and highly tolerant. Additional protective strategies involve activation of DNA repair systems (RecA/LexA), chaperones and proteases (GroEL, DnaK, ClpXP), stabilization of the cell envelope (LptD, BamA, Mla), and metabolic rerouting toward NADPH-producing pathways such as the pentose phosphate pathway (PPP). These adaptations collectively contribute to prolonged MDK_99_ values without changes in MIC, reflecting survival without resistance.

Mechanisms underlying bacterial tolerance to antimicrobial stress are illustrated in Figure 3.

Bacterial tolerance represents a multifactorial and reversible survival strategy that enables a subpopulation to endure antimicrobial treatment without exhibiting resistance [20,42,43]. Key contributors include metabolic inactivation and slowdown [46,48], dormancy and toxin–antitoxin systems [59,60,61,62,63], oxidative stress regulation [53,57], and the SOS response [71,72,73,74,75]. Tolerant cells often downregulate growth-related processes while activating protective mechanisms such as chaperones and proteases [78,79,80,81,82], DNA repair systems [71,72,73,74,75,76,77], antioxidant enzymes [40,53,54], stabilization of the cell envelope [84,85,86,87,88,89], and metabolic rerouting toward NADPH-generating pathways such as the pentose phosphate pathway (PPP) [54,90,91]. This phenotype can emerge in both planktonic and biofilm-associated communities [29,49,50] in response to various stressors including antibiotics and photodynamic treatment [6,16,17]. While overlapping with resilience and persistence, tolerance is defined by its transient, non-heritable nature and lack of MIC increase [20,42]. Further insights into the interplay between these adaptive responses remain essential for optimizing light-based and conventional antimicrobial strategies.

## 4. Mechanisms of Resilience

While tolerance allows bacteria to endure lethal stress by slowing growth or entering dormancy, resilience describes their ability to actively recover once the stressor is removed. This dynamic phenotype involves repair of cellular damage, metabolic reactivation, and phenotypic plasticity. Unlike passive survival, resilience entails reprogramming of core systems to restore homeostasis and resume replication, particularly under fluctuating or sublethal exposures, such as intermittent oxidative or photodynamic stress [22,92].

Key mechanisms involved in resilience include:

Genomic repair and replication restart: As discussed in Section 3, DNA integrity is essential for survival under oxidative or photodynamic stress. In resilience, repair systems such as RecA, RecBCD, and UmuDC [93] contribute not only to lesion clearance but also to replication restart once stress is lifted. This coordination, often linked to the SOS response, enables resumption of cell division and population regrowth. In *S. aureus*, *recA* knockout strains showed markedly impaired recovery after sublethal aPDI, underscoring its role in post-stress regrowth [77]. Similarly, in *Bacillus subtilis*, sublethal photodynamic exposure was reported to upregulate nucleotide excision and transcription-coupled repair genes (*uvrA*, *mfd*), consistent with a role in damage clearance and replication restart [94].

Proteostasis-driven recovery: Chaperone systems such as GroEL, DnaK, and the disaggregase ClpB, together with ATP-dependent proteases (e.g., ClpXP), are induced by oxidative and photodynamic stress. While tolerance relies on their buffering role to stabilize proteins, resilience specifically engages their recovery function: refolding damaged proteins and degrading irreversibly oxidized peptides. This active proteome repair restores cellular functionality and supports rapid post-stress regrowth [81,95].

Membrane repair and PMF restoration: Resilient cells mobilize membrane-associated systems including LptD, BamA, OmpF, and the Mla pathway to restore outer membrane integrity and lipid asymmetry after oxidative damage [96]. RpoS-dependent remodeling contributes to envelope reinforcement during recovery [97]. Crucially, restoration of the proton motive force (PMF)—particularly its ΔpH component—supplies the energy required for periplasmic protein folding, solute transport, and reactivation of metabolism [98]. Envelope stress response systems such as the Cpx two-component system and the σ^E regulon orchestrate these repair programs, highlighting resilience as an active recovery strategy distinct from passive survival [99].

Redox rebalancing and metabolic reboot: Unlike tolerance, which dampens metabolic activity to reduce ROS generation, resilience requires rapid reactivation of metabolic fluxes. During post-stress recovery, *Escherichia coli* reroutes carbon through the oxidative branch of the pentose phosphate pathway (PPP), increasing NADPH production to support antioxidant defenses and anabolic repair [98,100]. This shift is accompanied by suppression of NADH-generating pathways to limit further oxidative damage. In contrast to tolerance strategies based on metabolic dormancy and lag time extension [101], resilience reflects a dynamic return to redox homeostasis and biosynthetic activity.

Regulatory plasticity during recovery: Global regulators such as RpoS and SoxRS in Gram-negative bacteria, σ^B in Gram-positive species, and OxyR in both lineages are essential in managing the transition from damage control to recovery. While these regulators initiate stress survival programs and growth arrest during the stress exposure (see Section 3), they also promote the reactivation of repair, metabolism, and cell division pathways after stress resolution. This dual-phase functionality supports the broader concept of stress-induced resilience [102,103,104].

**Figure 4:**
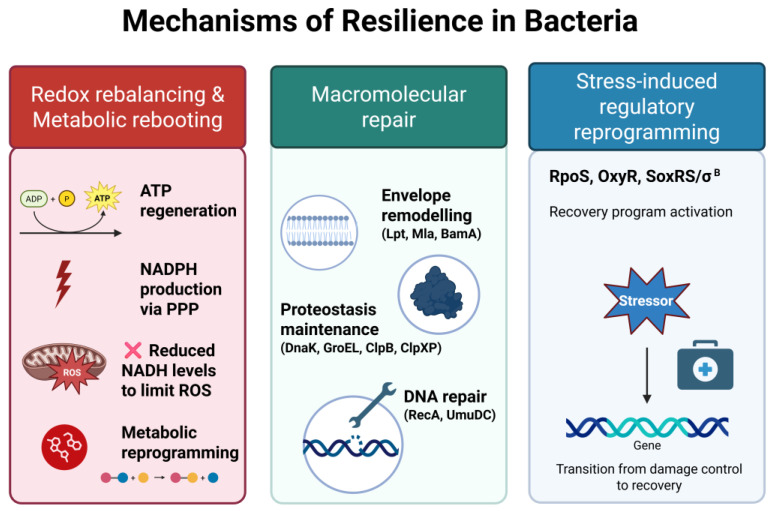
Mechanisms of bacterial resilience. Resilience reflects the ability of bacteria to recover from transient, non-lethal stress. This process involves (1) redox rebalancing and metabolic re-booting, including ATP regeneration, enhanced NADPH production via the pentose phosphate pathway (PPP), reduced NADH levels to limit ROSs generation, and global metabolic reprogramming; (2) macromolecular repair, encompassing envelope remodeling (e.g., Lpt, Mla, BamA), proteostasis maintenance (e.g., DnaK, GroEL, ClpB, ClpXP), and DNA repair (e.g., RecA, UmuDC); (3) stress-induced regulatory reprogramming, coordinated by global regulators such as RpoS, OxyR, SoxRS, and σ^B, which enable a transcriptional shift from damage control to recovery. Together, these mechanisms promote the restoration of physiological functions once stress is relieved.

Bacterial resilience represents an active and reversible recovery program, distinct from both tolerance and persistence. Instead of passively withstanding stress, resilient populations actively engage in redox rebalancing and metabolic rebooting, macromolecular repair, and regulatory reprogramming to restore physiological functions once the stressor is removed (Figure 4). These coordinated processes enable rapid resumption of growth after sublethal damage, whether triggered by antibiotics, oxidative agents, or photodynamic inactivation. Importantly, resilience does not involve stable genetic alterations or increased MIC values but rather reflects phenotypic plasticity and repair capacity [90,91,97,100,101,102]. Understanding the interplay between tolerance, persistence, and resilience is crucial for the optimization of antimicrobial strategies, particularly as light-based treatments may uniquely modulate recovery trajectories compared with conventional antibiotics.

## 5. Cross-Talk and Mechanistic Overlap

Although bacterial tolerance and resilience are defined as distinct survival strategies—one enabling persistence during stress, the other promoting recovery post-stress—they often rely on overlapping molecular mechanisms, including global regulators (e.g., RpoS, OxyR), chaperone systems (e.g., DnaK, GroEL, ClpB), and DNA repair pathways (e.g., RecA, UmuDC) [77,81,95,97,102]. These phenotypes may coexist or even transition into one another depending on the timing, intensity, and nature of the stressor [42,77,103]. In clonal populations exposed to sublethal photodynamic stress, such overlap may manifest as phenotypic heterogeneity, where dormant-like cells exhibiting delayed killing (tolerance) coexist alongside metabolically active cells capable of rapid recovery (resilience). This supports the view that tolerance and resilience are not discrete, mutually exclusive categories, but rather points along a mechanistic continuum shaped by environmental context and regulatory architecture.

**Figure 5:**
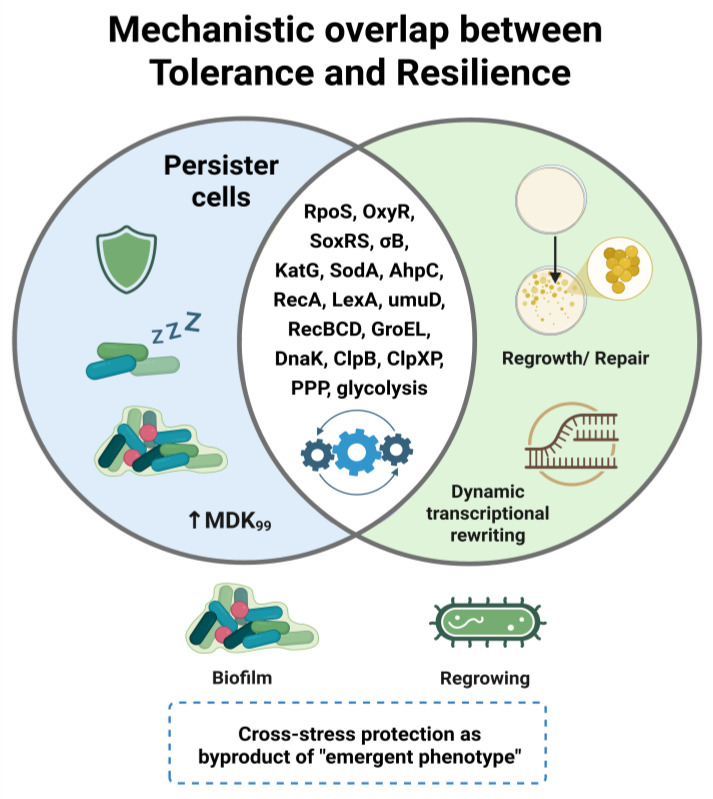
Mechanistic overlap between bacterial tolerance and resilience. Tolerance is characterized by persister formation, metabolic slowdown, and prolonged MDK_99_, whereas resilience reflects active recovery programs, including redox rebalancing, macromolecular repair, and transcriptional reprogramming. Shared regulators and stress-response pathways (e.g., RpoS, OxyR, SoxRS, σ^B; antioxidant enzymes; DNA repair; chaperones/proteases; PPP/glycolysis) highlight a mechanistic continuum rather than strict dichotomy. Cross-stress protection may emerge as a byproduct of this continuum, underscoring resilience as a clinically and environmentally relevant hidden threat.

Mechanistic overlap between tolerance and resilience therefore suggests emergent, context-dependent behaviors within bacterial populations. Cross-stress protection and phenotypic heterogeneity have been documented as emergent phenomena arising from shared regulatory and repair mechanisms [104]. Additionally, antibiotic persistence has been shown to emerge as a spatial and metabolic property in structured microbial communities [105]. This conceptual continuum and its mechanistic overlap are illustrated in Figure 5.

As summarized in Table 1, shared mechanisms and their differential roles in tolerance and resilience are outlined below.

## 6. Reinterpreting Phototreatment Outcomes: Is Resilience the Missing Piece?

Resilience appears particularly relevant in light-based antimicrobial approaches such as aBL and aPDI, where multiple cellular targets are simultaneously affected. Under these conditions, bacteria may not evolve resistance or tolerance in a classical sense but instead transiently adjust their physiology to survive and regrow once stress ends.

While most studies on aBL and aPDI focus on resistance or tolerance, some findings suggest alternative interpretations. For example, Leanse et al. described cycle-dependent drops in aBL efficacy against *Acinetobacter baumannii* during sublethal exposure (notably cycles 9, 16, and 17), which later reverted spontaneously [108]. Though interpreted as a lack of stable tolerance, these fluctuations may reflect resilience-linked recovery. Similarly, Zhang et al. observed a transient decline in *Candida albicans* susceptibility to aBL around cycles 4–5, followed by partial recovery and stabilization. While no heritable change was detected, this trend could reflect a short-lived physiological adjustment rather than true tolerance [109]. Amin et al. reported that *Pseudomonas aeruginosa* subjected to ten repeated aBL cycles showed overall stable killing, but with a short transient dip in efficacy around cycles 3−4 that was later restored, again consistent with resilience-like recovery [110]. By contrast, the Tomb study showed that *Staphylococcus aureus* exposed to repeated aBL cycles maintained stable susceptibility throughout, with no evidence of transient fluctuations or resilience-like behavior [111].

In contrast, other phototreatment studies revealed the emergence of stable tolerance. Our previous work demonstrated that *E. coli* exposed to repeated aBL developed a persistent shift in killing kinetics without enhanced regrowth, consistent with tolerance rather than resilience [17]. Our group also showed that *S. aureus* developed stable tolerance after ~20 cycles of RB-mediated aPDI [16]. Similarly, Pieranski et al. demonstrated that *Streptococcus agalactiae* subjected to repeated RB-mediated aPDI cycles acquired a stable tolerant phenotype supported by oxidative stress gene upregulation and altered physiology [112]. Snell et al. reported a comparable outcome for MB-mediated aPDI, including cross-tolerance with TBO [18].

Although definitive proof remains elusive, as shown in Table 2, these cases highlight the importance of distinguishing biological variability from transient, non-heritable recovery. Standard tolerance assays (e.g., MIC shifts, survival curves) may overlook resilience, underscoring the need for refined experimental frameworks to disentangle survival during stress from recovery post-stress.

## 7. Resilience, Tolerance, and Resistance in the Context of Oxidative Phototreatments

The increasing use of oxidative, light-based antimicrobial therapies demands a refined view of bacterial survival dynamics. While resistance is typically tracked via stable increases in MIC, tolerance and persistence often escape standard susceptibility readouts. Tolerance denotes delayed killing (↑MDK) without MIC change; persistence reflects survival of small, dormant subpopulations; resilience captures rapid functional recovery once stress ceases [20,42,43,90,97,100,101,102,103]. These phenotypes may coexist within a single population, complicating eradication strategies [44,75,77].

Moreover, phototreatment-induced oxidative stress can engage cross-stress responses that modulate survival under other conditions (e.g., heat, desiccation, disinfectants), with species- and context-dependent outcomes [26,27,28,29,30]. Notably, recent work in *E. coli* suggests that aBL does not promote cross-stress resistance under the tested conditions [31].

To mitigate these challenges, phototreatment protocols should minimize sublethal exposures and integrate kinetic readouts. Useful approaches include combining light with adjuvants (e.g., redox disruptors or antibiotics), quantifying MDK and growth-delay kinetics, and explicitly monitoring post-exposure recovery [20,43,110].

Distinguishing resistance, tolerance, and resilience is not merely conceptual: it directly informs the design of robust and evolutionarily stable regimens, particularly for light-based strategies whose primary mode of action is oxidative damage [20,42]. Determining whether survivors exhibit transient reactivation (resilience), stable shifts in killing dynamics without MIC change (tolerance), or heritable increases in MIC (resistance) is essential for durable control in clinical and industrial settings [20,42,90,97,100,101,102,103]. Notably, antibiotic studies have demonstrated that tolerance can act as an evolutionary stepping-stone toward resistance development [23], underscoring the importance of distinguishing stress-specific trajectories when evaluating the long-term efficacy of antimicrobial interventions.

**Figure 6:**
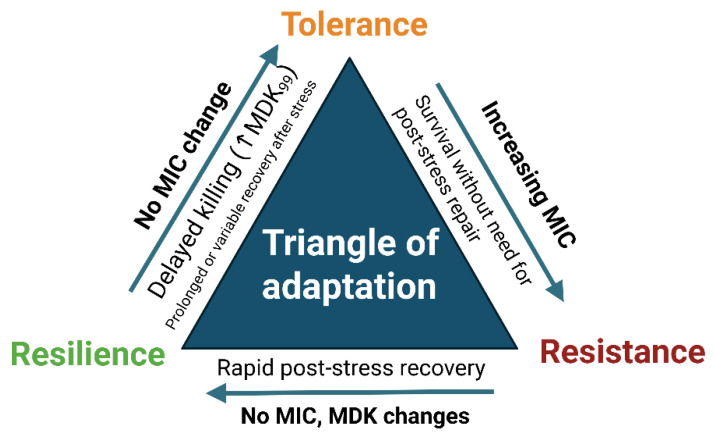
Triangle of adaptation. Conceptual framework distinguishing three bacterial survival strategies under oxidative or photonic stress: resistance (increased MIC with no requirement for post-stress repair), tolerance (delayed killing; increased MDK_99_ with unchanged MIC), and resilience (rapid functional recovery after treatment without lasting MIC/MDK changes). Arrows indicate the typical experimental readouts associated with each phenotype [20,42,43].

As a working model, survival strategies can be organized by their characteristic effects on MIC, MDK, and recovery, as illustrated in Figure 6:

Resistance—stable/elevated MIC with no recovery phase required (growth continues despite the presence of the stressor;

Tolerance—prolonged MDK without MIC increase;

Resilience—rapid post-stress regrowth without persistent MIC or MDK shifts [20,42,43,103].

Complementary experimental metrics to identify these traits are summarized in Table 3.

## 8. Materials and Methods

### Literature Search Strategy

Relevant literature was identified through systematic searches in Google Scholar, PubMed, and Scopus using combinations of keywords including “bacterial tolerance”, “resilience”, “oxidative stress”, “antimicrobial blue light”, “antimicrobial photodynamic inactivation”, and “reactive oxygen species”. To complement this process, the AI-supported tool Elicit (elicit.org) was occasionally tested to explore query formulation and prioritization. However, it was not used as a primary search engine, and all references included in this review were verified manually by the author to ensure accuracy and relevance. Additional references were obtained via citation chaining and expert knowledge of the field.

## 9. Conclusions and Future Perspectives

Phenotypic strategies such as tolerance and resilience are increasingly recognized as critical contributors to bacterial survival under oxidative and photodynamic stress. While often conflated, this review underscores their distinct biological logic: tolerance prolongs survival by delaying bacterial killing, whereas resilience enables rapid recovery following exposure. Although both lead to treatment failure in practice, their mechanistic divergence has important conceptual and therapeutic implications.

Despite their differences, these phenotypes often share overlapping regulatory pathways, such as RpoS and oxidative stress regulons, making it challenging to discriminate between them experimentally. The decisive factor may lie in stress dynamics and population heterogeneity, which determine whether slowed killing (tolerance) or accelerated regrowth (resilience) predominates. This highlights the need for assays capable of disentangling survival during exposure from recovery after stress removal, particularly under sublethal or intermittent treatment conditions. The possibility of cross-stress priming, whereby exposure to ROS enhances survival under unrelated stressors (e.g., heat, desiccation, biocides) further complicates the picture and warrants careful consideration in both clinical and industrial contexts.

In the context of light-based antimicrobials, such as aBL and aPDI, assessing immediate killing efficacy is insufficient. Preventing survival and recovery of stress-adapted subpopulations is essential to avoid regrowth, recurrence, and the potential emergence of resistance. For tolerance, promising therapeutic directions include strategies targeting persister cells, biofilm matrix disruption, or metabolic reprogramming (e.g., PMF disruptors, ROS-boosting adjuvants). For resilience, potential interventions may aim at blocking repair and recovery pathways, such as RecA inhibitors, chaperone modulators, or envelope stress-response inhibitors. Rational combinatorial strategies—pairing light-based treatments with such adjuvants—should be prioritized and validated under physiologically relevant and fluctuating conditions (e.g., biofilms, food contact surfaces, or infected tissues).

While multi-omics and single-cell approaches remain indispensable to map the regulatory circuitry of stress responses, the highest priority should be the establishment of standardized kinetic benchmarks that clearly distinguish tolerance (prolonged MDK) from resilience (shortened recovery time). Without such harmonized assays, cross-study comparison will remain elusive, and therapeutic implications will remain blurred. Integrating resilience-aware metrics into susceptibility testing would represent a paradigm shift: beyond MIC and MDK, recovery kinetics should become a routine readout when evaluating novel antimicrobials, particularly light-based interventions.

In conclusion, tolerance and resilience are not redundant concepts but complementary pieces of the bacterial survival puzzle. Their mechanistic overlap highlights shared stress-defense nodes, while their divergence emphasizes temporal and population-level dynamics. Addressing both strategies will be essential for the development of next-generation antimicrobial regimens that are not only bactericidal but also resilience-proof.

## Figures and Tables

**Table 1:** Shared mechanisms between bacterial tolerance and resilience.

Mechanism	Key Players	Role in Tolerance	Role in Resilience	References
**Global stress regulators**	RpoS, SoxRS, OxyR, σ^B	Dormancy, metabolic suppression, ROS mitigation	Repair coordination, redox balance, metabolic rebooting	[97,102,103]
**Antioxidant enzymes**	KatG, SodA/B, AhpC, Tpx, PerR (Gram+)	ROSs scavenging during stress exposure	Redox balance maintenance during recovery	[32,40,41,57,58]
**DNA repair (SOS response)**	RecA, LexA, UmuD, RecBCD	Genome stabilization under stress, mutagenesis	Replication restart and DNA repair after stress removal	[71,72,73,74,75,76,77,93]
**Chaperones and proteases**	GroEL, DnaK, ClpB, ClpXP, Lon, IbpA/B, Hsp33	Prevention of protein aggregation	Protein refolding and proteome recovery	[78,79,80,81,82,83]
**Efflux and envelope systems**	Efflux: AcrAB–TolC, NorA, MexAB–OprM; Envelope: LptD, BamA, OmpF, Mla system	Reduction of intracellular toxic load, envelope stabilization	Outer membrane repair, PMF restoration	[55,56,85,86,87,96,99,106,107]
**Metabolic adaptation**	PPP, NADPH/NADH balance, glycolytic rerouting	Metabolic slowdown to reduce ROS formation	PPP upregulation for NADPH supply and repair	[53,54,90,91,98,100]
**Persister/dormancy formation**	(p)ppGpp, TA systems, RpoS	Entry into persistence, multidrug tolerance	Persister awakening and division restart	[20,42,43,44,45,46,47,48,49,50,51,52,59,60,61,62,63,64,65,66,67,68,69,70]

Abbreviations: NADH—nicotinamide adenine dinucleotide (reduced form); NADPH—nicotinamide adenine dinucleotide phosphate (reduced form); PMF—proton motive force; PPP—pentose phosphate pathway; (p)ppGpp—guanosine tetraphosphate/pentaphosphate; TA systems—toxin–antitoxin systems; σ^B—sigma factor B.

**Table 2:** Re-evaluation of selected aBL studies in the context of bacterial resilience.

Species	Treatment	Observations	Original Interpretation	Possible Resilience Indicator	Reference
***A. baumannii***	aBL (10 repeated cycles)	Transient drop in efficacy at cycles 9, 16, and 17; full susceptibility restored in later cycles	No tolerance detected	Cycle-dependent fluctuation in killing efficacy; may reflect resilience-driven recovery	[108]
***C. albicans***	aBL (10 cycles)	Slight, temporary efficacy reduction at cycles 4–5; later cycles showed stable or increased susceptibility	No tolerance detected	Short-lived decline in susceptibility; possible physiological adjustment without heritable change	[109]
***P. aeruginosa***	aBL (10 cycles)	Overall stable killing across cycles, but transient reduction in efficacy at ~cycles 3–4, later restored	No tolerance detected	Short-lived dip in susceptibility may indicate resilience-type recovery	[110]
***S. aureus***	aBL (15 cycles)	Stable efficacy across all cycles; no cumulative loss observed	No tolerance detected	None detected; findings argue against resilience under repeated aBL stress.	[111]
***E. coli***	aBL (15 cycles)	Sustained delay in killing kinetics across repeated exposures; effect persisted even after subculturing	Stable tolerance detected	Tolerance rather than resilience; stability observed across passages	[17]
***S. aureus***	RB-mediated aPDI (20 cycles)	Progressive reduction in efficacy across cycles; stable tolerant phenotype maintained even after subculturing	Stable tolerance detected	Genetic alterations supporting long-term tolerance; not resilience	[16]
***S. aureus***	MB-mediated aPDI (7 cycles)	Progressive loss of efficacy; stable tolerant phenotype emerged. Cross-tolerance observed	Stable tolerance detected	Cross-tolerance across structurally related photosensitizers (MB and TBO) indicates genetic adaptation	[18]
***Streptococcus agalactiae***	RB-mediated aPDI (10 cycles)	Stable reduction in efficacy; tolerance persisted after 5 passages without selection	Stable tolerance detected	Genetic/physiological adaptations (↑ oxidative stress genes, SCVs, ↓ PS uptake)	[112]

Abbreviations: aBL—antimicrobial Blue Light; aPDI—antimicrobial Photodynamic Inactivation; RB—rose bengal; MB—methylene blue; TBO—toluidine blue O.

**Table 3:** Diagnostic parameters proposed to distinguish resistance, tolerance, and resilience based on MIC shifts, MDK dynamics, and post-treatment regrowth behavior. These measurements complement standard susceptibility assays and reveal phenotypes often undetectable by MIC alone.

Parameter	Description	Proposed Method	Distinguishes From	References
**Recovery time** **after treatment**	Time to reach OD_600_ threshold or CFU rebound after a defined exposure	Growth-curve monitoring (OD/time)/regrowth CFU	**Tolerance** (where the main change occurs during exposure, i.e., altered MDK)	[20,110]
**Transient stress-response expression**	Upregulation of oxidative/damage-response genes immediately after exposure that normalizes upon recovery	qPCR/RNA-seq at 0 h and post-aBL/aPDI timepoints	**Resistance** (constitutive expression, usually associated with stable genetic changes)	[16,25,82,102] *
**MDK drift** **without** **MIC change**	Shifts in MDK_50_ or MDK_90_ across cycles, while MIC remains stable	Time-kill assays with regrowth analysis	**Resistance** (MIC↑); **Resilience** (no persistent MDK shift after cycles)	[43,76,104]
**Fluctuating** **efficacy in** **repeated cycles**	Non-monotonic log_10_-kill changes that revert to baseline in later cycles	Cycle-by-cycle log-kill plots with recovery tracking	Indicates **resilience** if recovery occurs without persistent MDK shift across passages	[15,109,110]

* Transcriptomic profiling to distinguish transient from stable stress responses remains an open research area; Abbreviations: CFU—colony forming units; MDK—minimum duration for killing; MIC—minimum inhibitory concentration; OD600—optical density at 600 nm. ↑ indicates an increase.

## Data Availability

No new data was created.

## Abbreviations

The following abbreviations are used in this manuscript:

aBL	antimicrobial blue light
aPDI	antimicrobial photodynamic inactivation
CFU	colony-forming unit
MB	methylene blue
MDK	minimal duration of killing
MIC	minimal inhibitory concentration
NADH	nicotinamide adenine dinucleotide (reduced form)
NADPH	nicotinamide adenine dinucleotide phosphate (reduced form);
OD	optical density
PMF	proton motive force
PPP	pentose phosphate pathway
(p)ppGpp	guanosine tetraphosphate/pentaphosphate
qPCR	quantitative polymerase chain reaction
RB	rose bengal
RNS	reactive nitrogen species
ROS	reactive oxygen species
RNA-seq	RNA sequencing
TBO	toluidine blue O
